# ASO Authors Reflections: Patient Age and Survival After Surgery for Esophageal Cancer

**DOI:** 10.1245/s10434-020-08661-w

**Published:** 2020-05-29

**Authors:** Giola Santoni, Jesper Lagergren, Matteo Bottai

**Affiliations:** 1 Upper Gastrointestinal Surgery, Department of Molecular Medicine and Surgery, Karolinska Institutet, Karolinska University Hospital, Stockholm, Sweden; 2 School of Cancer and Pharmaceutical Sciences, King’s College London, London, UK; 3 Division of Biostatistics, Institute of Environmental Medicine, Karolinska Institutet, Stockholm, Sweden

## Past

Curatively intended esophagectomy, currently the most effective treatment for locally advanced esophageal cancer, is an extensive procedure with high risks of serious and sometimes lethal complications.^1^ The short-term safety and long-term benefits of esophagectomy in older people are controversial. The decision as to whether to recommend esophagectomy or not in older patients may be better informed if the patient’s risk of mortality after surgery is available. One key measure is the change over time of the probability of dying and how this may differ across age groups. This study aimed to quantify this probability by using a novel statistical method, the event-probability regression.^2^^,^^3^ This method can estimate the mortality risk, properly defined as the probability of dying at any given time point for those who are still alive at that point (working paper: http://www.imm.ki.se/biostatistics/eventprob/Working_paper_2020.pdf). This estimated risk is bounded between zero and one, and can model odds ratios of mortality as a function of time and patients’ characteristics, such as age. Event-probability regression can be easily estimated with the Stata command “stpreg” available at http://www.imm.ki.se/biostatistics/eventprob.

## Present

This nationwide and population-based cohort study included 1731 patients who underwent curatively intended esophagectomy between 1987 and 2010 in Sweden.^4^ The patients were followed for at least 5 years and date of death, when this occurred, was recorded. Patient data included age, attained education level, comorbidity, and tumor characteristics.

The probability of mortality increased with age and decreased with time after surgery. Forty-year-old patients alive at 6 months had a probability of dying of 0.3 patients/year, while in 80-year-old patients the probability was 0.5 patients/year. At 4 years after esophagectomy, these probabilities declined to 0.07 patients/year and 0.14 patients/year, respectively. At any time, the odds of dying for 80-year-old patients were 2.1 (95% CI 1.56–2.76) times that of 40-year-old patients.

## Future

The study suggests that age is an independent risk factor for worse survival both in the short- and long-term after esophageal cancer surgery, which may be considered in the clinical decision-making.

The statistical method used in this paper allowed curves to be obtained for the probability of dying throughout the follow-up time. This method can be applied to any study where the aim is to analyze the time to an event of interest, such as diagnosis of cancer or onset of a disease. Traditionally, in this type of study the analysis focuses on hazard ratios and hazard functions. The hazard function, however, does not represent a probability. Proper probabilities and their odds ratios should be preferred by medical professionals when assessing the chance of survival in patients. Because obtaining probabilities and their odds ratios is simple with standard computer software, we recommend calculating and reporting these instead of the hazards.^5^
